# Jianpi Qinghua Fomula alleviates insulin resistance via restraining of MAPK pathway to suppress inflammation of the small intestine in DIO mice

**DOI:** 10.1186/s12906-022-03595-0

**Published:** 2022-05-09

**Authors:** Yahua Liu, Xu Han, Mengjie Cai, Shenyi Jin, Zihui Yan, Hao Lu, Qingguang Chen

**Affiliations:** 1 Department of Endocrinology, Shuguang Hospital Affiliated to Shanghai University of Traditional Chinese Medicine, Shanghai, 201203 China; 2 Diabetes Institute, Shuguang Hospital Affiliated to Shanghai University of Traditional Chinese Medicine, Shanghai, 201203 China; 3 Shanghai Key Laboratory of Traditional Chinese Clinical Medicine, Shanghai University of Traditional Chinese Medicine, Shanghai, 201203 China

**Keywords:** insulin resistance, Jianpi qinghua fomula, MAPK pathway, Inflammation of small intestine, Glucose and lipid absorption

## Abstract

**Background:**

Jianpi Qinghua Fomula (JPQHF), a clinically proven prescription,has been applied to cure insulin resistance(IR) and type 2 diabetes (T2DM) for more than 20 years. Here, we will unravel the underlying molecular mechanisms relevant to the therapeutic actions of JPQHF.

**Methods:**

High-fat(HF)diet-induced obesity(DIO)mouse were established in our research, along with insulin resistance. After the administration of JPQHF 5 or 6 weeks, the parameters of the glucose and lipid metabolism were measured. Flow cytometry and Luminex were utilized to assess the inflammation in small intestine,whilst Western blot was used to determine the relative expression levels of the MAPK pathway-related proteins. The glucose and lipid transporter of small intestine was assessed by immunofluorescence and ELISA, and the expression of insulin signaling pathway was detected by Western blot.

**Results:**

The metabolic phenotypes of DIO mouse were ameliorated after 6-week oral administration of JPQHF; Meanwhile,JPQHF downregulated levels of IL-1β,IL-6, TNF-α and IFN-γ but upregulated the ratio of M2/M1 macrophages in the small intestine. The elevated expressions of p-P38 MAPK/P38 MAPK、p-JNK/JNK and p-ERK1/2/ERK1/2 were reversed by JPQHF. Moreover, JPQHF enhanced expression of PI3K,p-AKT/AKT, p-IRS1/ IRS1, p-IRS2/ IRS2 and apoB48 in small intestine, and facilitated the translocation of GLUT2 to the basal side of small intestine epithelial cells.

**Conclusion:**

JPQHF alleviates insulin resistance in DIO mice, and this effect may be associated with its restraining of inflammation of small intestine via attenuating MAPK pathway, and then diminishes small intestinal glucose and lipid absorption.

**Supplementary Information:**

The online version contains supplementary material available at 10.1186/s12906-022-03595-0.

## Background

Type 2 diabetes is a grim health burden on a global scale. Obesity-induced insulin resistance is implicated as the initiating factor for type 2 diabetes, which can accelerate the progression to various metabolic disorders and cardiovascular diseases [1], wherefore ameliorating insulin resistance is becoming an urgent need.

Chronic state of low-grade inflammation is the pivotal pathological basis of obesity and insulin resistance. High-fat-diet-induced inflammatory pathological shift in the intestine occur prior to the development of obesity [2–4], accompanied by macrophages in intestinal lamina propria producing pro-inflammatory cytokines [5, 6]. The energy imbalance in which energy intake exceeds dissipation is considered as the foremost cause of obesity-induced insulin resistance. As the main organ of glycolipid absorption, the small intestine has essential roles in the pathogenesis of obesity-induced insulin resistance [3, 7]. Intestinal inflammation impaires epithelial insulin signaling pathway which in turn triggers glycolipid intake disorder [7, 8], accumulates excessive amounts of glucose and free fatty acids(FFA)in the circulation and exacerbates systemic insulin resistance [9–11].Consequently, intestinal immune system may be a novel therapeutic target in the treatment of insulin resistance.

Recently, accumulating evidence has shown the preventive and curative effect of traditional Chinese medicine (TCM) in T2DM and associated complications [12, 13]. The JPQHF is adapted from ‘Bupiwei Xieyinhuo Shengyang Decoction’ which derived from the < Theory of Spleen and Stomach > . JPQHF has been applied in the treatment of T2DM and insulin resistance in Shuguang Hospital Affiliated to Shanghai University of Traditional Chinese Medicine( Shanghai, China) for more than two decades. JPQHF enhances metabolism of lipids and improves insulin sensitivity [14–16], but the potential pharmacodynamic mechanism is still remains incompletely clear. Therefore, the aim of our research was to investigate weather the JPQHF would improve insulin resistance via ameliorating intestinal inflammation.

## Material and method

### Preparation of JPQHF

The JPQHF consists of Codonopsis pilosula (Franch.) Nannf (15 g), Astragalus membranaceus (Fisch.) Bunge (15 g), Dioscoreae rhizoma(15 g), Polygonatum sibiricum (15 g),Coptis chinensis Franch (3 g), Scutellaria baicalensis Georgi (9 g), Radix Puerariae (15 g) and Euonymus alatus (Thunb.) Sieb (15 g). The herbs were obtained from Lei-Yun-Shang Pharmacy(Shanghai,China) and were identified by Dr Guanglin Xu from Pharmacy Department of Shuguang Hospital Affiliated to Shanghai University of Traditional Chinese Medicine. The herbs fulfilled the standard requirements of the 2015 version of the Chinese Pharmacopoeia.

JPQHF was prepared by Pharmacy Department of Shuguang Hospital Affiliated to Shanghai University of Traditional Chinese Medicine. All above eight herbs were submersed in 1000 ml of distilled water and extracted in a ceramic clay pot at 100 ℃ for 30 min with continuously stirred twice. The mixed extract was concentrated at 100 ℃ for 30 min and centrifuged at 13,000 rpm for 30 min at 4 ℃.Then the supernatant was filtered through a sieve until each milliliter contains 1.5 g drug.

### Animal groups and treatment

Six-week-old healthy male C57BL/6 mice weighted 18 ± 2 g were acquired from Beijing Weitong Lihua Laboratory Animal Technology Co., Ltd. Shanghai Branch, (Shanghai, China), certification no. SCXK(HU)2017–0011. Mice were housed under controlled conditions: 12 h light/12 h dark cycle, ambient temperature of 23 ± 3 °C and humidity 55 ± 15%. All experimental procedures involving animals were approved by the Animal Experiment Ethics Committee of Shanghai University of Traditional Chinese Medicine, and the approval no.PZSHUTCM 190,823,004.

The mice were randomly divided into normal group (NOR) and the high-fat diet group, and were fed a normal diet (1,010,009; Nanjing Synergy Biology Co., Ltd.; Nanjing, Jiangsu province, China) or the HFD containing 60 kJ % fat (D12492; Research Diets, New Brunswick, NJ, USA) respectively for 12 weeks. After continuous feeding of 12 weeks, the weight of mice in the high-fat diet group was 1.2 times of the normal group were considered as DIO mice. The DIO mice were then divided into three groups according to body weight via SPSS 25.0 (SPSS Inc., Chicago, USA): DIO group (DIO), Metformin group (MET) and JPQHF group (JPQH),and daily treated with oral normal saline, metformin suspension and JPQHF individually. The metformin tablets (Merck Serono, Geneva, Switzerland) was dissolved in physiological saline at a density of 300 mg/kg body weight.

### Metabolic phenotypes correlated parameters analysis

The body weight of mice was measured by the same electronic scale at the same time every week (Saturday morning 9:00–11:00).

After administration of 5 weeks, both IPGTT and IPITT were performed after 12 h of fasting. Glucose(1.5 g/kg) or insulin(0.5U/10 g,D134876A,Eli Lilly and Company, Indianapolis, Indiana, U.S.) was administered intraperitoneally respectively. Glucose levels were monitored by glucometer (ACCU-CHEK Active, Roche, Mannheim, Germany) via sampling from the end of the caudal vein at 0, 30, 60, 90 and 120 min after glucose or insulin injection. Finally the area under the curve (AUC) was calculated by the trapezoidal rule.

Levels of serum fasting insulin(FINS) were determined by ELISA(CSB-E05071 m, Wuhan Huamei Biological Engineering Co., Ltd., Wuhan, Hubei province, China).The cholesterol (TC), triglycerides (TG), high density lipoprotein cholesterol (HDL-C), and low density lipoprotein cholesterol (LDL-C) concentration was tested by the fully automated biochemical analyzer(7020, Hitachi High-Tech, Tokyo, Japan).

### Morphological analyses

The small intestine was separated and fixed in 2% neutral buffered formalin for 8 h, then transferred to 20% glucose, 30% glucose, and embedded with OCT. The tissue specimens were applied to analysis the transposition of GLUT2 using the anti-GLUT2 antibody (Ab54460,1:200, Cambridge, UK) by fluorescence microscope (80i, Nikon, Tokyo, Japan).

### Western Blotting

Total protein was extracted from small intestine and quantified by BCA assay in accordance with the manufacturers protocol (23,225, Thermo Fisher Scientific, MA, USA). Immunoblotting was performed as described previously [17]. The primary antibodies p-P38 MAPK (4613S,1:1000), P38 MAPK (9212S,1:1000), p-JNK (4668S, 1:1000), JNK (9252S,1:1000), p-ERK1/2(4370S,1:1000), ERK1/2 (4695S,1:1000), PI3K (4249S,1:1000), Akt(3063S,1:1000), p-Akt (4060S, 1:1000), IRS1 (2382S,1:10 00), p-IRS1 (3203S,1:1000), p-IRS2 (4502S,1:1000) and secondary antibody were purchased from Cell Signaling Technology (MA, USA).Chemiluminescent images were acquired by imaging system (5200, Tonan, Shanghai, China) and analyzed using the Protein Array Analyzer plugin for ImageJ.

### Enzyme-linked Immunosorbent Assays (ELISA)

Concentrations of apoB48 in small intestinal tissue in serum were evaluated by ELISA (CSB-E16506m, Wuhan Huamei Biological Engineering Co., Ltd., Wuhan, Hubei province, China) according to kit instructions. The OD value was read at 450 nm by a microplate (ELX800, Biotek Instruments, Vermont, USA) after incubation at 37 °C for 20 min.

### Flow cytometry

Separation and digestion of small intestinal tissue were operated under the guidance of instructions(Lamina Propria Dissociation Kit, mouse,130–097-410, Miltenyi Biotec, Bergisch Gladbach, Germany). Intestinal immune cell single cell suspension was placed in blank, compensation control and sample tube. The corresponding cell surface fluorescent antibody was added in compensation control tube, meanwhile, Fc Block, Fixable Viability Stain 510 and cell-surface fluorescent antibodies such as CD45 (557, 659), F4/80 (565, 411), CD11b (552, 850), CD206 (565 250) and CD86 (553,692) which were purchused from Becton, Dickinson and Company (New Jersey, USA) were put into sample tube in turn. Of note, incubating for 10 min at 4 °C in the dark and washing by FBS were needed after the reagent was added at above step. The flow cytometer (FACS Canto II, Becton, Dickinson and Company, New Jersey, USA) and software flow cytometer (V10, Becton, Dickinson and Company, New Jersey, USA) was utilized to detect and analysis the ratio of immune cells in the lamina propria of the small intestine.

### Luminex

Total protein was extracted from small intestine and quantified by BCA assay in adherence to the the manufacturer,s protocol (23,225,Thermo Fisher Scientific, MA, USA). The standard and premixed beads mixture were diluted according to the method provided in the instructions (MTH17MAG-47 K,MILLIPLEX,Merck KGaA, Darmstadt, Germany). 25 μl of distilled premixed beads mixture and 25 μl of sample were added to sample well, while 25 μl standard and 25 μl of cell clesolution were put in each standard well.The 96-well plate was incubated at room temperature for 2 h on the shaker and then was placed on magnetic stand. Biotin-labeled antibody complex and diluted streptavidin-labeled PE were put in each well sequentially. Finally, the beads was detected by Luminex(X-200, Luminex Corporation, Texas, USA). The concentration ratio of cytokine to sample was applied to statistics.

### Statistical analysis

All data were expressed as the means ± SD. One-way ANOVA test was applied for the comparison between groups using SPSS 25.0 (SPSS Inc., Chicago, USA). *P* < 0.05 was considered to indicate statistical significance.

## Results

### JPQHF ameliorated metabolic phenotype in DIO mice

**Fig. 1:**
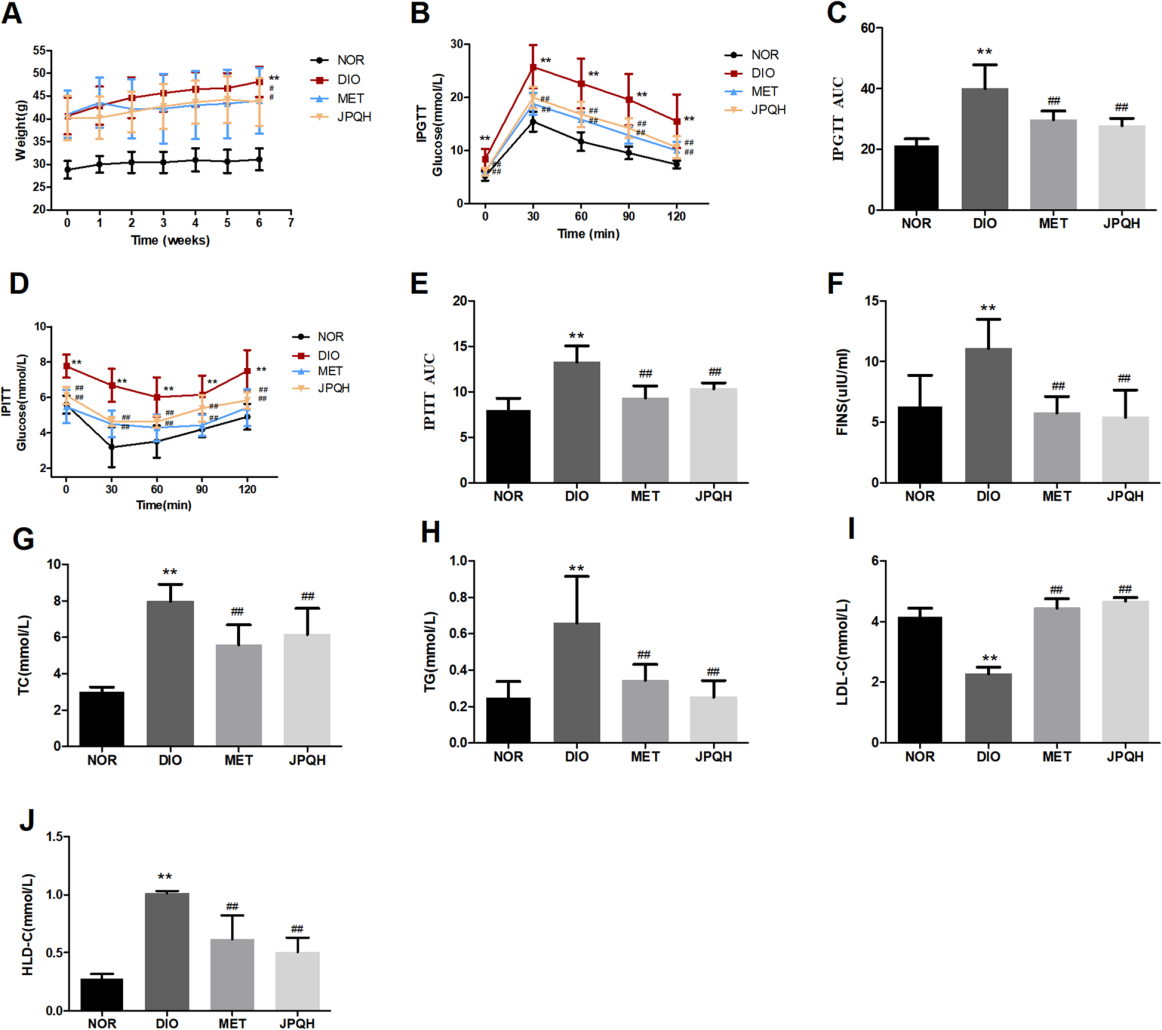
JPQHF ameliorated metabolic phenotype in DIO mice. **A** Body weight were measured weekly after JPQHF administration. **B-C** IPGTT and (**D-E**) IPPTT after 5 weeks’treatment of JPQHF. **F** fasting insulin (FINS), (**G**) cholesterol (TC), (**H**) triglycerides (TG), (**I**) low density lipoprotein cholesterol (LDL-C) and (**J**) high density lipoprotein cholesterol (HDL-C) concentration were detected at the 6th week. (*n* = 6) Values are mean ± SD. NOR: normal control; DIO: HFD induced obesity mouse; MET: metformin group(DIO mouse treated with metformin at a dose of 300 mg/kg/d; JPQH: DIO mice treated with JPQHF(20.961 g/kg/d). *P < 0.05 vs. the NOR group; **P < 0.01 vs. the NOR group; #*P* < 0.05 vs. the DIO group; ##*P* < 0.01 vs. the DIO group

To elucidate the contribution of JPQHF to metabolic phenotype, the DIO mouse model was utilized in this study. As shown in Fig. 1, the DIO mouse expressed elevated body weight, blood glucose at all time-point and AUC of IPGTT and IPITT, FINS, TC, TG, LDL-C and decreased HDL-C, which indicated the attack of hyperglycemia, hyperinsulinemia, insulin resistance and lipid metabolism disorder. After JPQHF intervention for 6 weeks, these damages was improved significantly. The JPQHF diminished the body weight (*P* < 0.05), blood glucose at various time points and AUC of IPGTT (*P* < 0.01) and IPITT (*P* < 0.01), FINS (*P* < 0.01), TC (*P* < 0.01), TG (*P* < 0.01), LDL-C (*P* < 0.01) and enhanced HDL-C (*P* < 0.01). Consistent with above findings, these results collectively corroborated that JPQHF ameliorated insulin resistance with glucose and lipid metabolism.

### JPQHF attenuated inflammation of small intestine in DIO mice

**Fig. 2:**
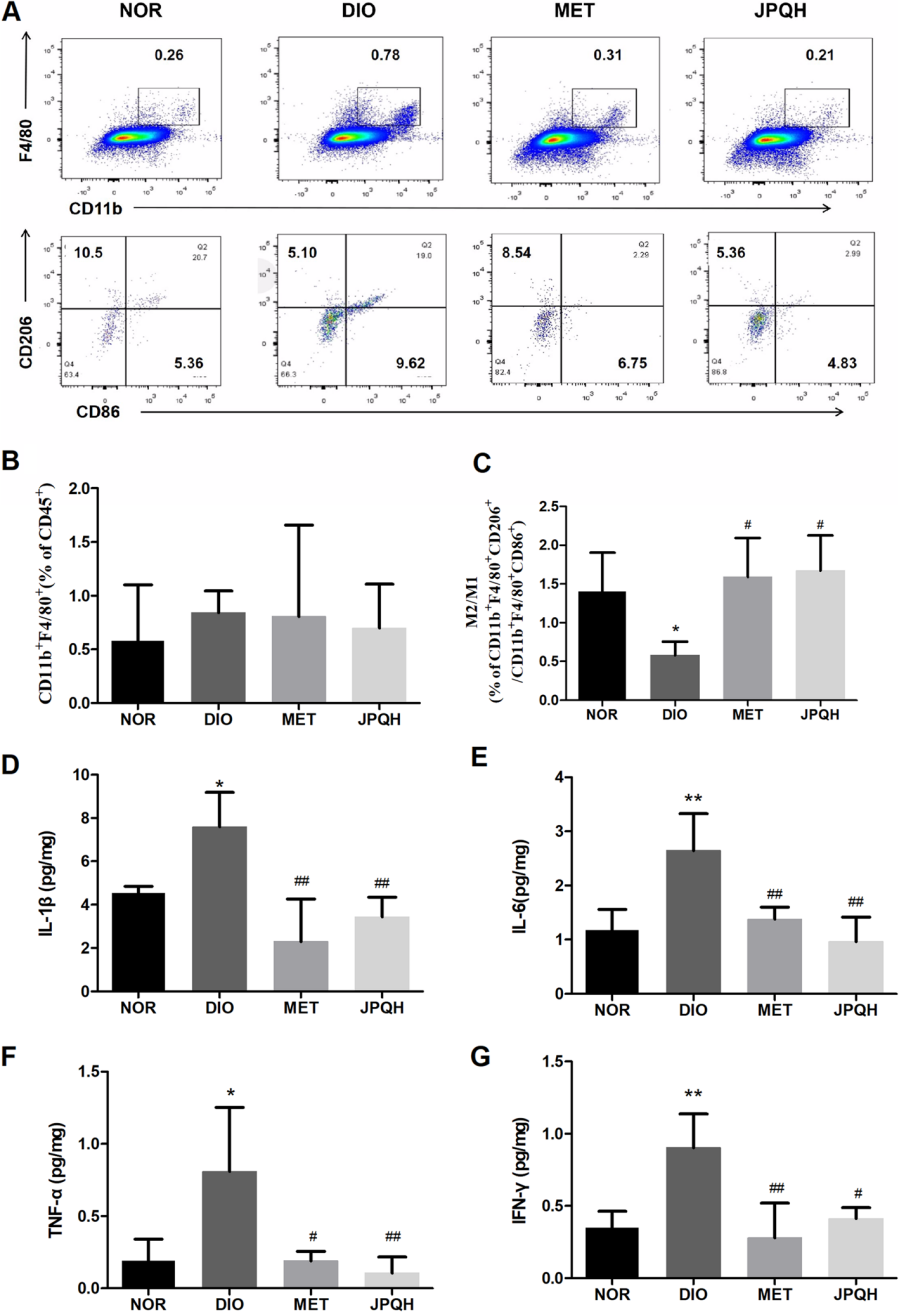
JPQHF attenuated inflammation of small intestine in DIO mice. **A-C** Flow cytometry analyzed the proportion of macrophage and the ratios of M2/M1 macrophage. Luminex evaluated the density of (**D**) IL-1β, (**E**) IL-6, (**F**) TNF-α, (**G**) IFN-γ (*n* = 6) of small intestine. Values are mean ± SD. *P < 0.05 vs. the NOR group; **P < 0.01 vs. the NOR group; #*P* < 0.05 vs. the DIO group; ##*P* < 0.01 vs. the DIO group

Obesity-related intestinal inflammation is accompanied by the increased production of pro-inflammatory mediators and less anti-inflammatory cytokines [4, 18, 19]. In this study, we discussed the role of JPQHF in intestinal inflammation. As it can be discerned from the Fig. 2, the the proportion of macrophage (CD45^+^CD11b^+^ F4/80^+^) exhibited no comparable difference (*P* > 0.05), conversely, the ratio of M2 macrophage(CD45^+^CD11b^+^ F4/80^+^CD206^+^)/M1 macrophage(CD45^+^CD11b^+^ F4/80^+^ CD86^+^) was significantly elevated (*P* < 0.05). Additionally, the pro-inflammatory cytokines such as IL-1β,IL-6, TNF-α and IFN-γ were increased. The above results implied that high-fat diet feeding resulted in intestinal inflammation. Reciprocally, JPQHF prominently shifted the macrophage polarization toward a higher M2/M1 ratio(*P* < 0.01) critically, lessened the levels of IL-1β (*P* < 0.01), IL-6 (*P* < 0.01), TNF-α (*P* < 0.01) and IFN-γ (*P* < 0.05). Accordingly, the JPQHF attenuated the pro -gression of intestinal inflammation of DIO mice.

### JPQHF decreased MAPK activation in the small intestine of DIO mice

**Fig. 3:**
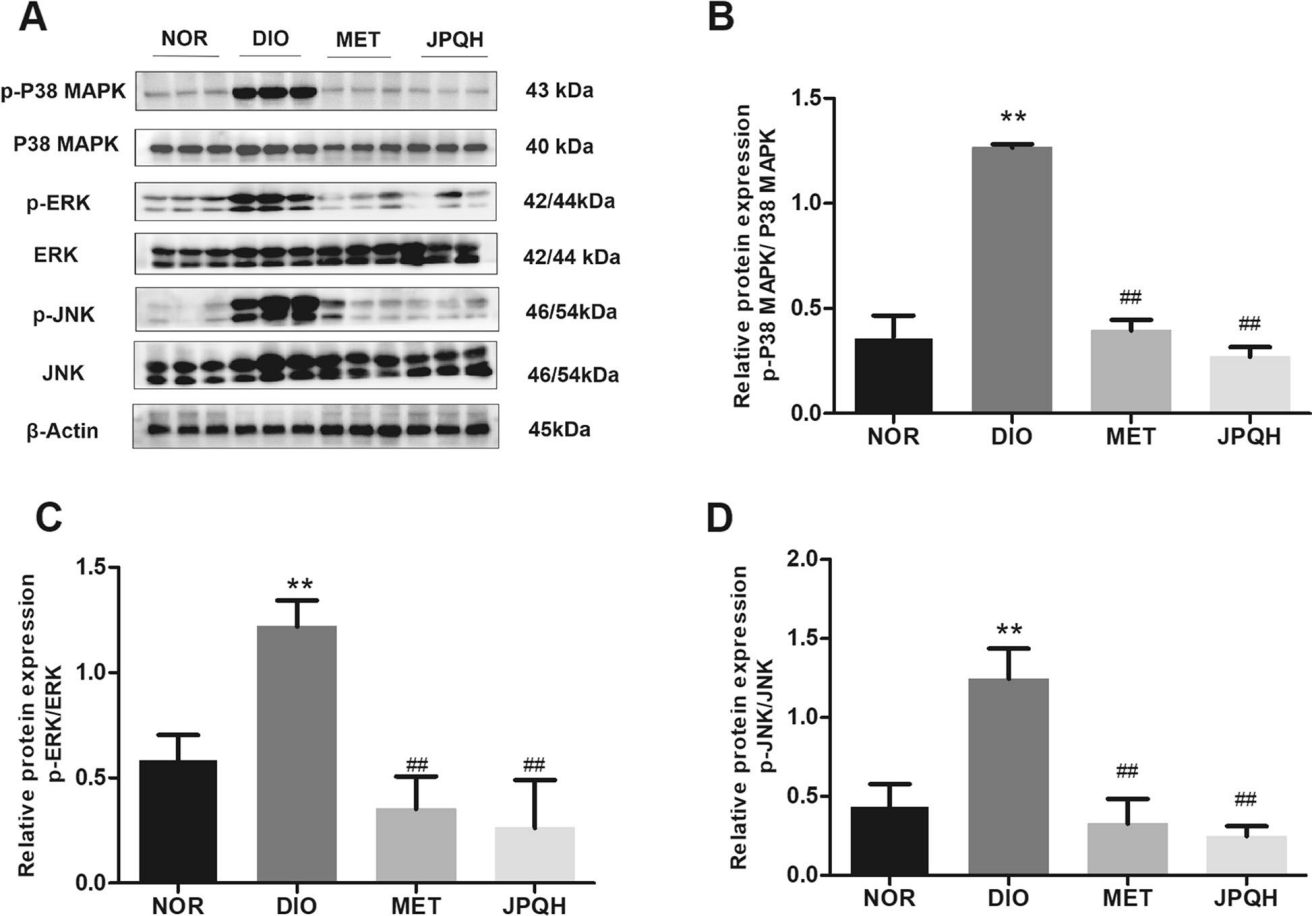
JPQHF decreased MAPK activation in the small intestine of DIO mice. **A **Western blot observed the expression of (**B**) p-P38 MAPK/P38MAPK, (**C**) p-ERK/ERK and (**D**) p-JNK/JNK (*n* = 6). Values are mean ± SD. **P < 0.01 vs. the NOR group; ##*P* < 0.01 vs. the DIO group

MAPK signaling pathway plays an important role in macrophage activation, polarization and proliferation, which is the key regulator of intestinal inflammatory diseases simultaneously [20–22]. As presented in the Fig. 3, the JPQHF inhibited the activation of MAPK by suppressing of the ratio of p-P38 MAPK/P38 MAPK (*P* < 0.01)、p-JNK/JNK (*P* < 0.01) and p-ERK1/2/ERK1/2(*P* < 0.01) in small intestine tissue of DIO mice.

### JPQHF diminished glucose and lipid absorption in the small intestine of DIO mice

**Fig. 4:**
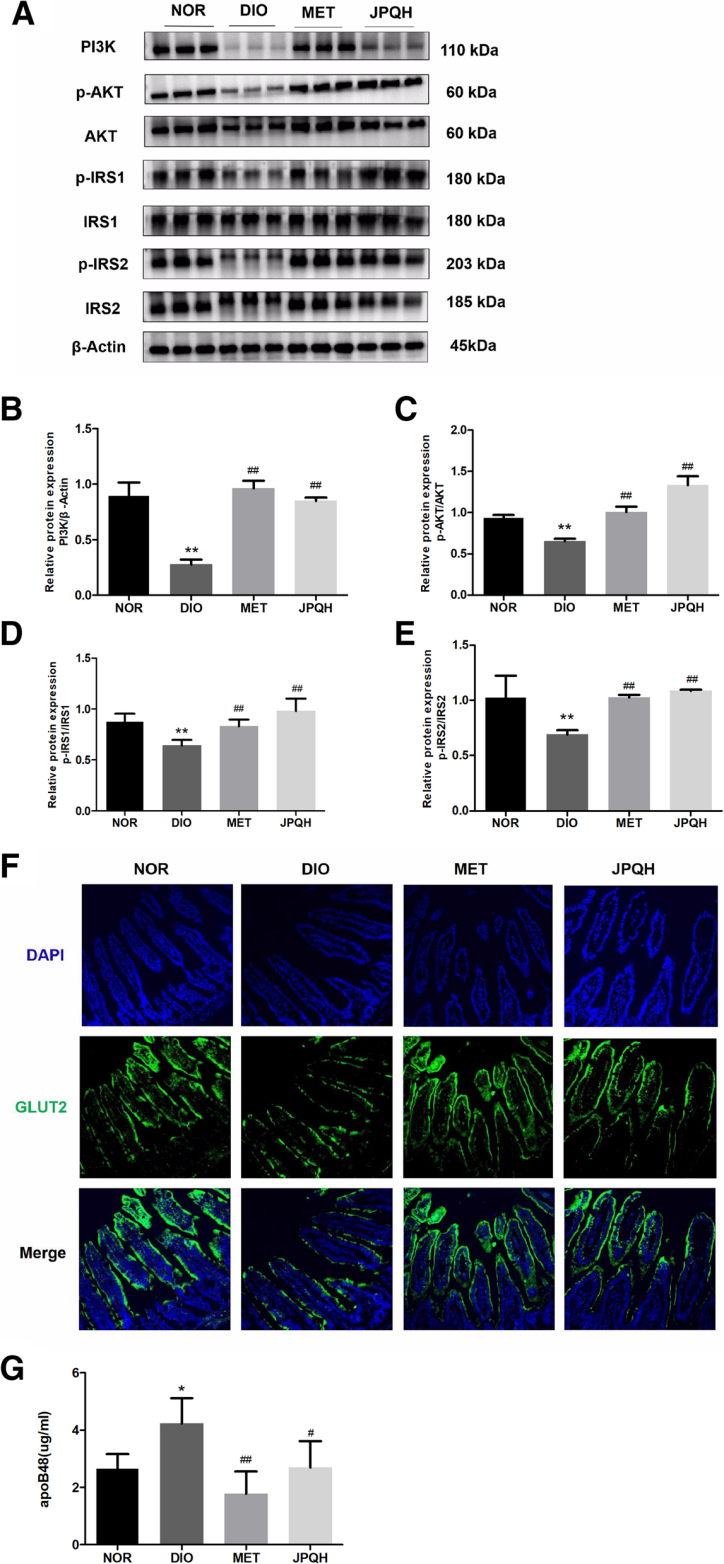
JPQHF diminished glucose and lipid absorption in the small intestine of DIO mice. **A **Western blot quantified the levels of (**B**) PI3K, (**C**) p-AKT/AKT, (**D**) p-IRS1/IRS1 and (**E**) p-IRS2/IRS2. **F** Immunofluorescence inspected the transpo -sition of GLUT2. 200 × magnification. (**G**) Serum concentration of apoB48. (*n* = 6) Values are mean ± SD. **P < 0.01 vs. the NOR group; ##*P* < 0.01 vs. the DIO group

Pro-inflammatory cytokines are pivotal factors of disrupting insulin signaling which interferes nutrient absorption in the small intestine through its impact on glucose and lipid transporters, which are essential for the uptake of glucose and lipid. This, in turn, lead to insulin resistance [23–25]. From Fig. 4 it is clear that JPQHF diminished the expression of PI3K (*P* < 0.01) and the the ratio of p-AKT/AKT (*P* < 0.01), p-IRS1/IRS1(*P* < 0.01), p-IRS2/ IRS2 (*P* < 0.01) considerably upregulated in DIO mice. The JPQHF lowered the concentration of apoB48 in small intestine (*P* < 0.01) and facilitated the translocation of GLUT2 from the brush border to the basal side of small intestine epithelial cells. Together, the JPQHF reduced glucose and lipid absorption of small intestine predominantly via IRS/PI3K/Akt activation.

## Discussion

Low-grade chronic systemic inflammation is the underlying etiological reason for insulin resistance. The latest investigations reconfirmed the notion that abnormal composition of small intestinal immune system triggers the local intestinal inflammation, which in turn contributes to systemic inflammation in other downstream insulin-sensitive organ [2–4]. Of note, macrophage have been considered pivotal in adipose which as key role in the inflammatory process associated with metabolic disease [26, 27]. Intestinal inflammation precedes incidence of systemic metabolic disorders in diet-induced obesity [18, 28, 29]. The obesity in humans is characterized by gut inflammation as shown by accumulation of pro-inflammatory intestinal macrophages [6]. The high-fat diet (HFD) increases inflammatory intestinal macrophages [5] and increased pro-inflammatory macrophages in the gut [2]. This is similar to what has been described in IBD and suggests disrupted differentiation of intestinal macrophages towards an anti-inflammatory/resident state. In addition, MAPK signaling pathway is thought to be important in macrophages activation, polarization and proliferation, which is considered pivotal in the occur of intestinal inflammatory diseases [20–22]. In the present study, we found that JPQHF could prominently ameliorates insulin resistance, shifted the macrophage polarization toward a higher M2/M1 ratio and reduced the inflammation factors expression remarkably in intestine,whilst inhibited the activation of MAPK. We infer that JPQHF improves inflammation in small intestine tissue via suppressing MAPK signaling pathway,in turn mitigates insulin resistance.

The small intestine is the primary site for nutrient absorption by glucose and lipids transporters. Facilitated diffusion of GLUT2 are involved in the transmembrane carriage of glucose [30, 31]. In the case of obesity and insulin resistance, overabundance of glucose is translocated to epithelial cells substrata and feeded into the circulation, which may account for postprandial hyperglycemia of insulin resistance and diabetes patient [10, 32]. Dietary fat is absorbed into small intestinal epithelial cells and is packaged into chylomicron, furthermore, the gut-specific apolipoprotein apoB48 is considered as a crucial component of the chylomicron [33, 34]. Serum apoB48 in a fasting state is intimately associated with higher postprandial lipemia, hence, it is now recognized as an important independent marker of postprandial hyperlipidemia risk [35, 36]. Perturbed insulin signaling pathway, the PI3K/Akt pathway, in intestinal cells will initiate the excessive intestinal glucose and lipid uptake. Impaired phosphorylation of insulin receptor substrates 1, 2(IRS1,2) and Akt in intestinal epithelial cells of obese patient facilitates GLUT2 localizing to the brush border [7]. Additionally, the activation of PI3K increases GLUT2 transcription [8]. Alternatively, blunted insulin signaling pathways is a state in which insulin unable to inhibit apoB48 synthesis. Furthermore, in the metabolic inflammation state, pro-inflammatory cytokines (e.g. IFN-γ,TNF-α,IL-6 and IL-1β) have been suggested to play a causal role in insulin signaling pathways suppression [22–25]. Our results revealed that JPQHF lowered small intestinal pro-inflammatory cytokines secretion, raised the expression of PI3K and phosphorylated IRS1, IRS2, Akt, facilitated the dislodgment of GLUT2 and depressed serum apoB48 concentrations. Hence, we conjecture that JPQHF reactivates insulin signaling pathways may through relieving of intestinal inflammation, eventually results in mitigating levels of glucose and lipid.

## Conclusions

**Fig. 5:**
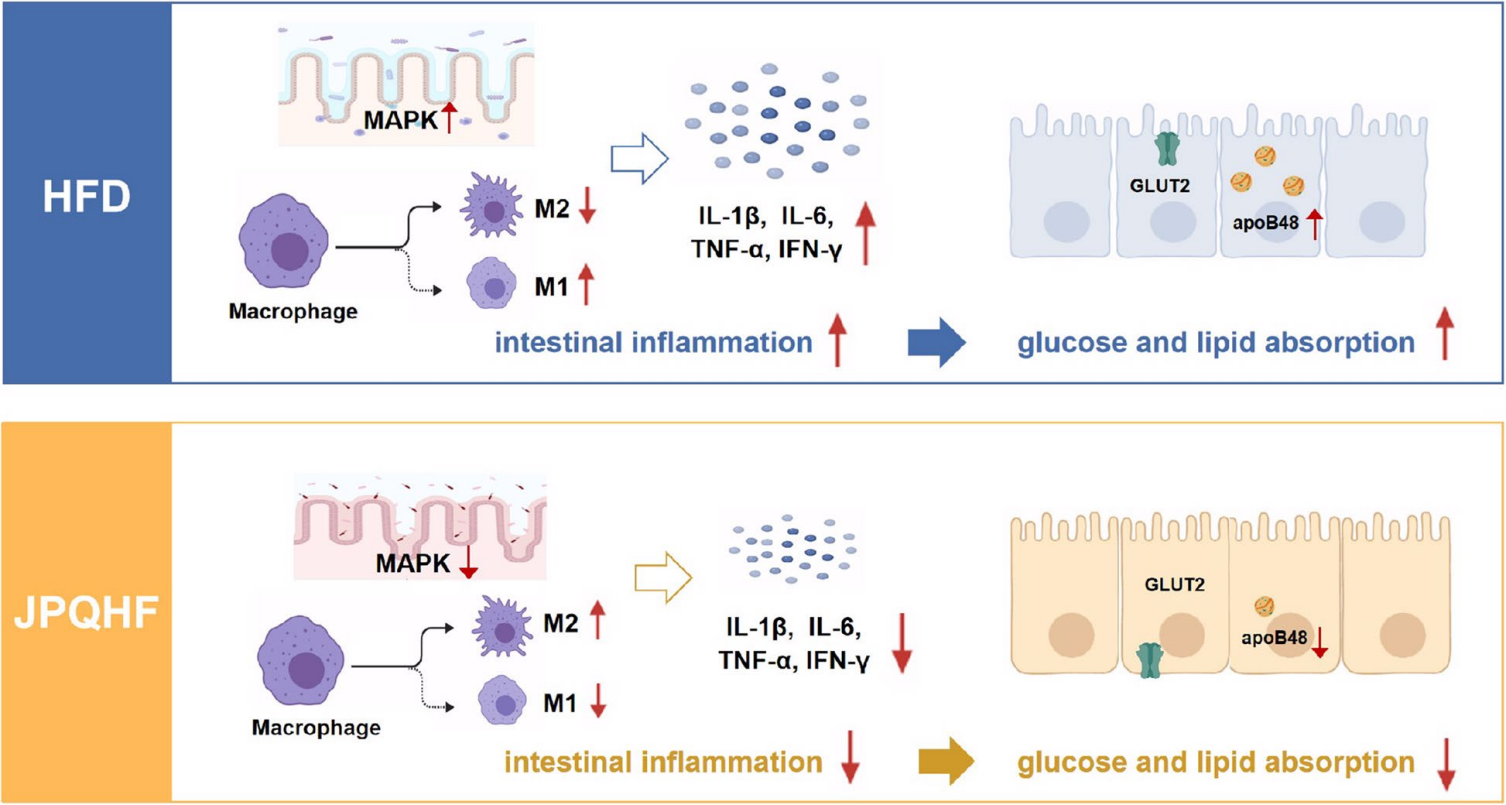
The mechanism of JPQHF attenuates insulin resistance

Generally, we demonstrate that JPQHF ameliorates insulin resistance in DIO mice,the speculated action mechanism as shown in Fig. 5. JPQHF suppresses intestinal inflammation,then diminishes glucose and lipid absorption,which eventually attenuates insulin resistance.

## Supplementary Information

**Additional file 1.** 

## Data Availability

All supporting data for this manuscript are in this article and supplementary Material. For further information, please contact the corresponding author.

## Abbreviations

DIO: Diet-Induced Obesity
HFD: High Fat Diet
T2DM: Type 2 Diabetes Mellitus
IR: Insulin Resistance
TCM: Traditional Chinese Medicine
MET: Metformin
MAPK: Mitogen-Activated Protein Kinase
P38 MAPK: P38 Mitogen-Activated Protein Kinase
ERK1/2: Extracellular Regulated Protein Kinases
JNK: C-Jun N-terminal Kinase
TC: Cholesterol
TG: Triglycerides
HDL-C: High Density Lipoprotein Cholesterol
LDL-C: Low Density Lipoprotein Cholesterol
PI3K: Phosphatidylinositol-3 Kinase
Akt: Protein kinase B
IRS: Insulin Receptor Substrate
apoB48: Apolipoprotein B 48
GLUT2: Glucose Transporter type 2
