# Supplementary material for: Jianpi Qinghua Fomula alleviates insulin resistance via restraining of MAPK pathway to suppress inflammation of the small intestine in DIO mice
Source: BMC Complement Med Ther. 2022 May 9;22:129. doi: 10.1186/s12906-022-03595-0 (PMC9088054; doi:10.1186/s12906-022-03595-0)
Supplement: Supplementary file 1 — Additional file 1. [file 12906_2022_3595_MOESM1_ESM.pdf]

Supplementary Information

Jianpi Qinghua Fomula Alleviates Insulin Resistance via Restraining of MAPK Pathway to Suppress Inflammation of the Small Intestine in DIO mice

Authors Explanation:

We guarantee that the given western blot was cropped from the same full-length blot. During the experiment, we stained the PVDF membrane with Ponceau red after protein transfer. In order to reduce the volume of reagents and antibodies required for the experiment, we removed the irrelevant regions of the target protein, and then used the target protein antibody for hybridization. However, the gel itself is never spliced. We only crop the captured image to present the region of interest.

Here we have presented the Original images of all blots .

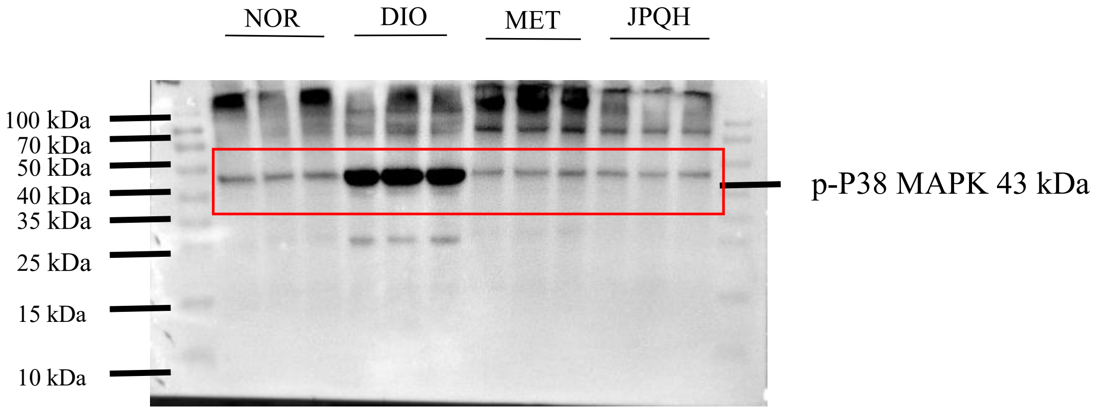

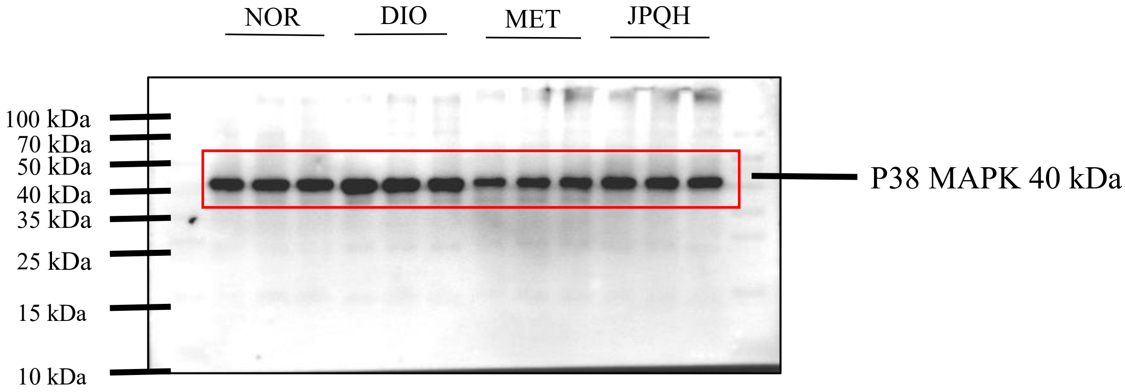

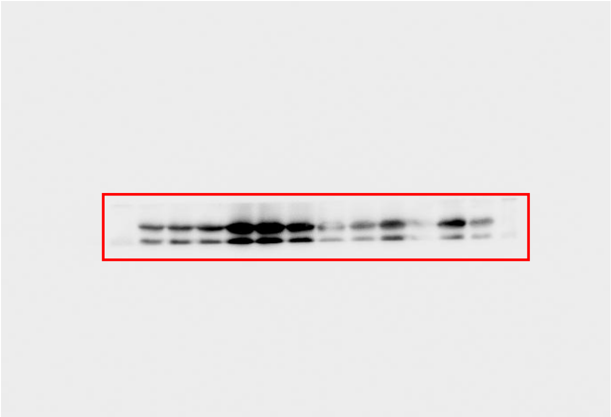

p-ERK 42/44 kDa

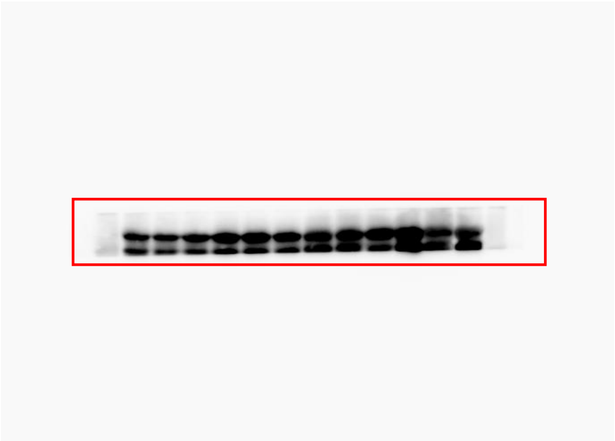

ERK 42/44 kDa

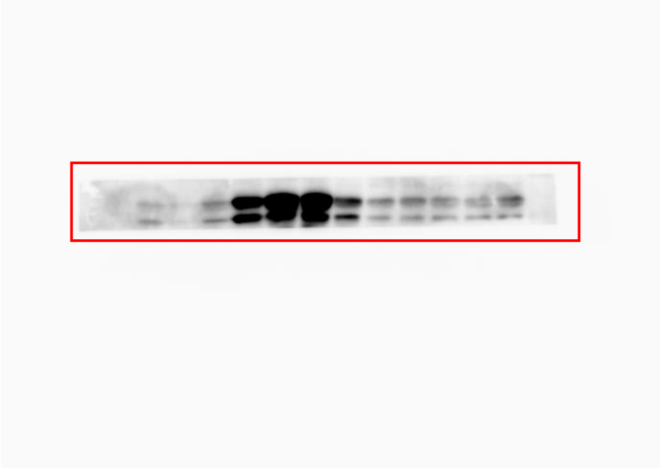

p-JNK 46/54 kDa

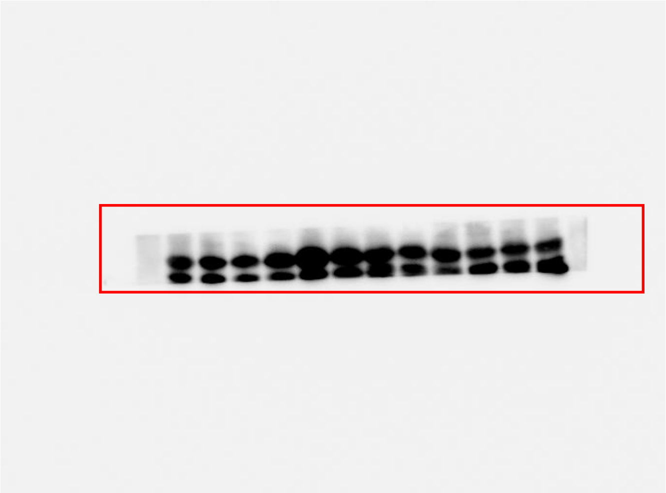

JNK 46/54 kDa

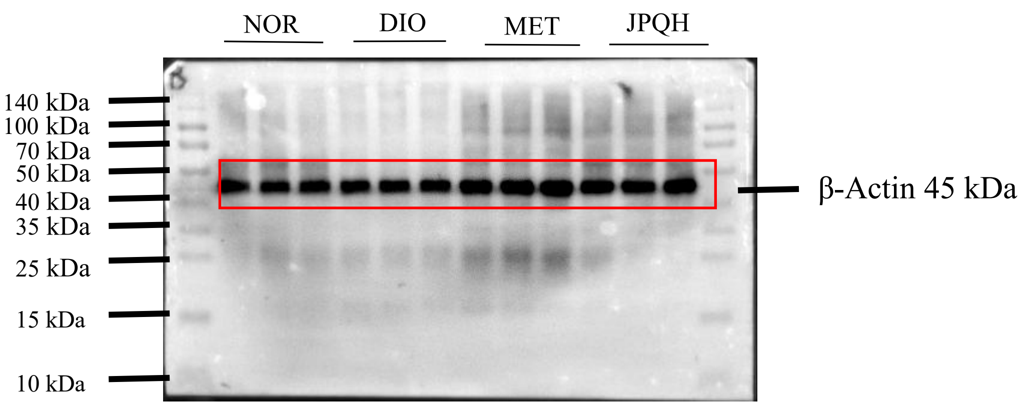
NOR
DIO
MET
JPQH
140 kDa
100 kDa
70 kDa
50 kDa
40 kDa
35 kDa
25 kDa
15 kDa
10 kDa
β-Actin 45 kDa

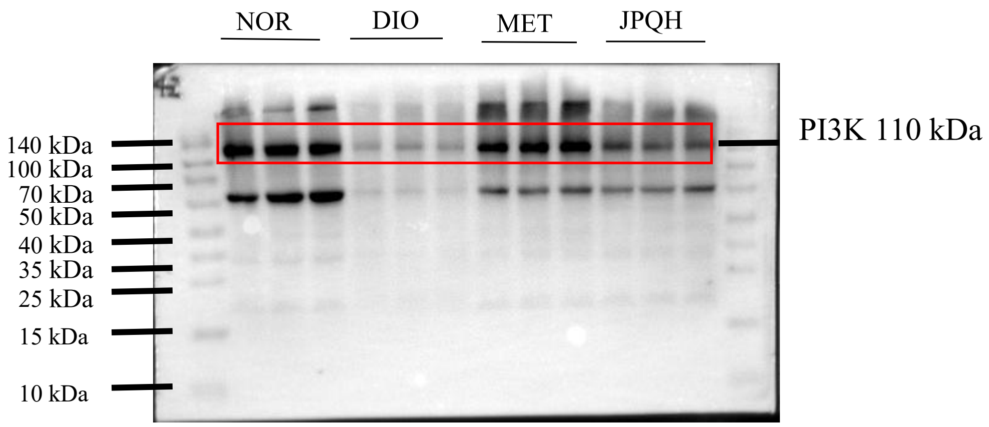
NOR
DIO
MET
JPQH
140 kDa
100 kDa
70 kDa
50 kDa
40 kDa
35 kDa
25 kDa
15 kDa
10 kDa
PI3K 110 kDa

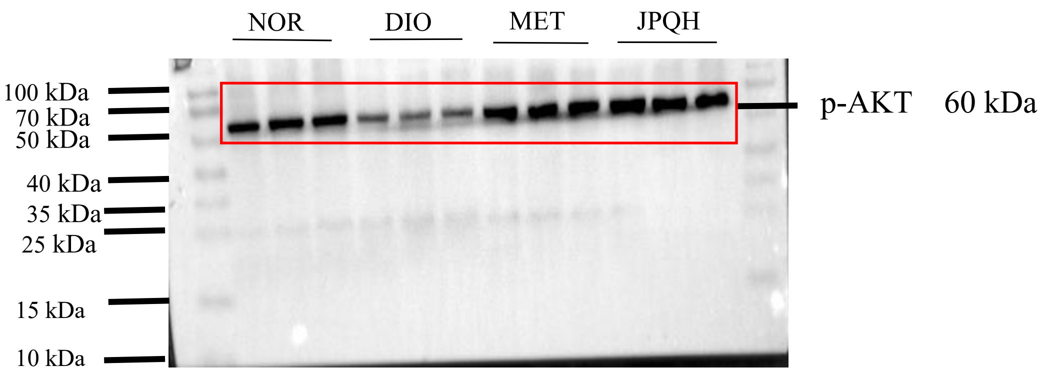
NOR
DIO
MET
JPQH
100 kDa
70 kDa
50 kDa
40 kDa
35 kDa
25 kDa
15 kDa
10 kDa
p-AKT 60 kDa

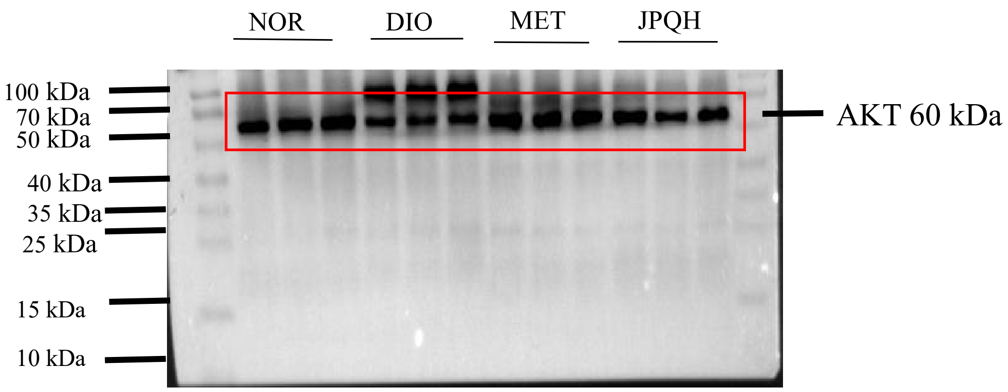
NOR
DIO
MET
JPQH
100 kDa
70 kDa
50 kDa
40 kDa
35 kDa
25 kDa
15 kDa
10 kDa
AKT 60 kDa

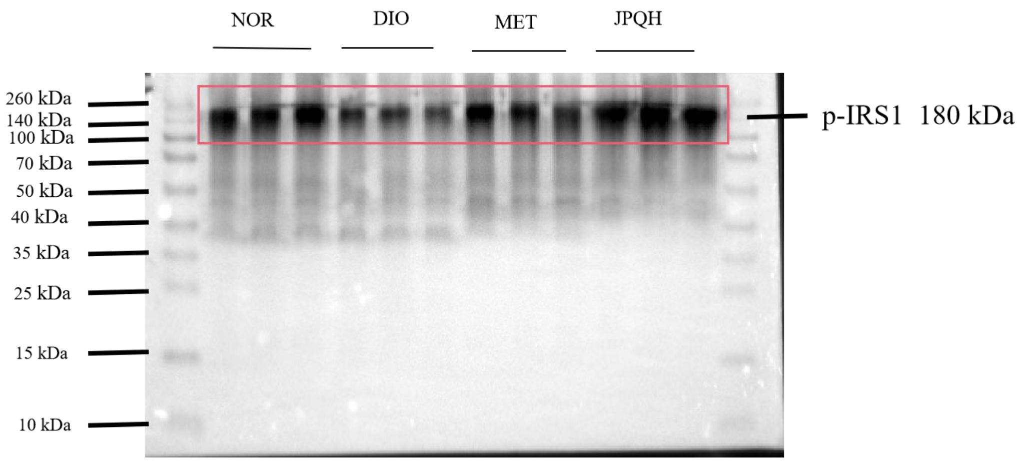
NOR
DIO
MET
JPQH
260 kDa
140 kDa
100 kDa
70 kDa
50 kDa
40 kDa
35 kDa
25 kDa
15 kDa
10 kDa
p-IRS1 180 kDa

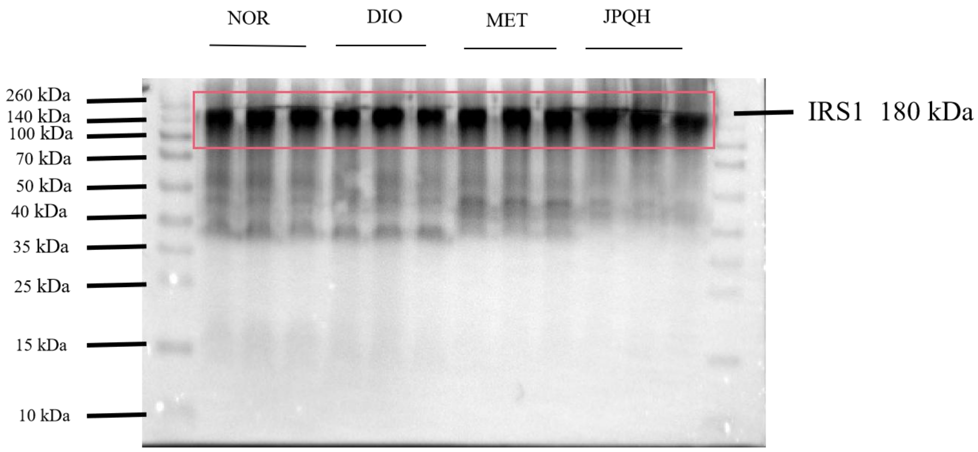
NOR
DIO
MET
JPQH
260 kDa
140 kDa
100 kDa
70 kDa
50 kDa
40 kDa
35 kDa
25 kDa
15 kDa
10 kDa
IRS1 180 kDa

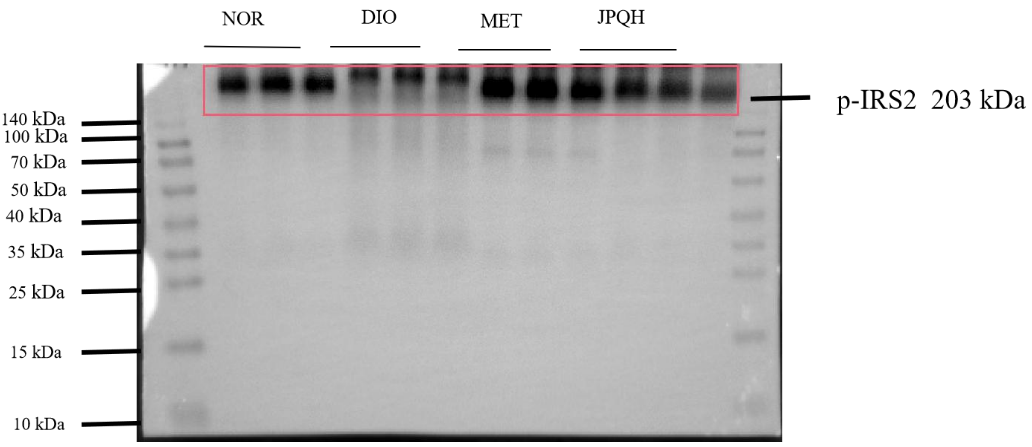
NOR
DIO
MET
JPQH
140 kDa
100 kDa
70 kDa
50 kDa
40 kDa
35 kDa
25 kDa
15 kDa
10 kDa
p-IRS2 203 kDa

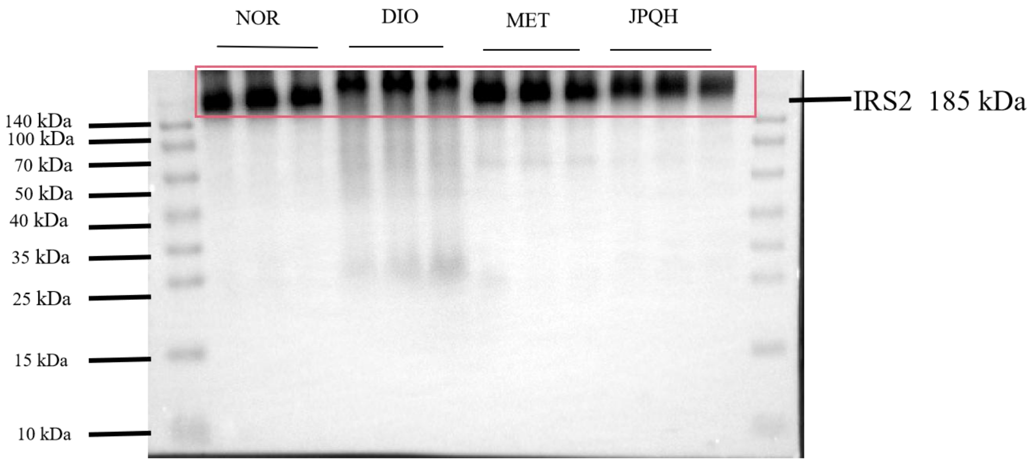
NOR
DIO
MET
JPQH
140 kDa
100 kDa
70 kDa
50 kDa
40 kDa
35 kDa
25 kDa
15 kDa
10 kDa
IRS2 185 kDa

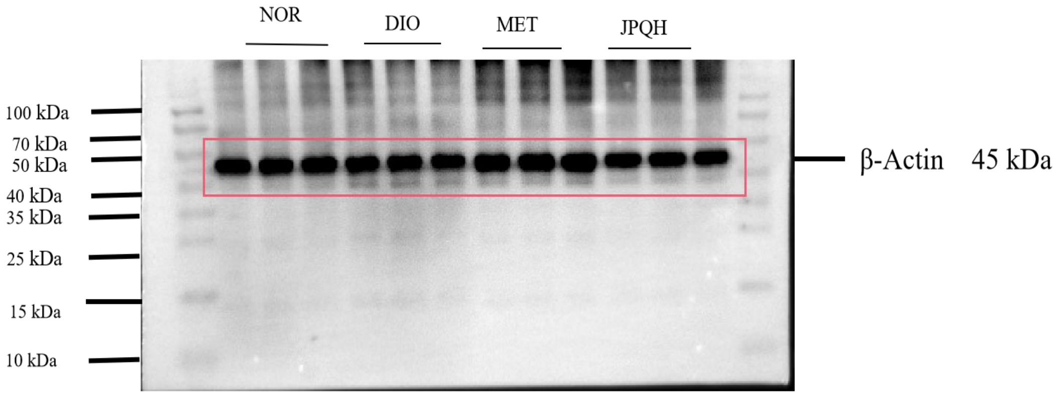
NOR
DIO
MET
JPQH
100 kDa
70 kDa
50 kDa
40 kDa
35 kDa
25 kDa
15 kDa
10 kDa
β-Actin 45 kDa
